# Supplementary material for: An epigenomic investigation of atrial fibrillation in a matched left and right atrial human cohort
Source: Clin Epigenetics. 2026 Mar 29;18:52. doi: 10.1186/s13148-026-02076-2 (PMC13045105; doi:10.1186/s13148-026-02076-2)

**SUPPLEMENTAL INFORMATION**

**Integrated epigenomic analysis in a paired left and right atrial fibrillation cohort**Rodriguez et al. 2025

**_______________________________________________________________**

**SUPPLEMENTAL DATA**

**Figure S1.** **Experimental design summary and extended details on H3K27ac profiling** (related to Figure 1) **(page 3)**

**Figure S2.** **Methodological details and extended properties for H3K27ac epigenomic regions enriched across sample groups** (related to Figure 2) **(p. 5)**

**Figure S3.** **Additional analyses on H3K27ac-enriched regions across sample groups (overlap with transcription factor motifs, binding events and cardiovascular GWAS variants)** (related to Figure 2) **(p. 7)**

**Figure S4.** **Gene expression re-analysis and extended details on candidate loci identified from integration of epigenomic and transcriptomic signals** (related to Figure 3) **(p. 9)**

**Figure S5.** **Extended details and analyses of candidate loci in an independent replication cohort** (related to Figures 4 and 5) **(p. 11)**

**Figure S6** **EWAS results and additional properties of DMRs across SR and AF samples** (related to Figure 6) **(p. 13)**

**Figure S7. Additional details and analyses for DMRs and their overlap with transcription factor motifs, binding events and GWAS variants** (related to Figure 7) **(p. 15)**

**Table S1**: Sample and patient details, including known clinical variables, operations performed and experimental readouts obtained in each sample. [SupplementalTable_S1.xlsx]

**Table S2.** Differentially-enriched regions with standard methods (DiffBind, TCseq). [SupplementalTable_S2.xlsx]

**Table S3**. Genomic regions enriched in H3K27ac across the four sample groups, and in AF vs SR samples. [SupplementalTable_S3.xlsx]

**Table S4.** Differentially-expressed genes across anatomical sides, disease status, or in each of the four sample groups. [SupplementalTable_S4.xlsx]

**Table S5**. Candidate loci with hallmarks of altered gene regulation in atrial fibrillation patients. [SupplementalTable_S5.xlsx]

**Table S6**. Enrichment of candidate loci among genes with dysregulated expression across atrial fibrillation patient cohorts. [SupplementalTable_S6.xlsx]

**Table S7**. Candidate loci selected for validation in an independent replication cohort with locus-specific quantitative PCR. **(p. 17)**

**Table S8**. Full results of ChIP-QPCR targeted validation across two independent amplicons for each candidate region [SupplementaryTable_S8.xlsx]

**Table S9**. Single CpG and DMR regions showing DNA methylation differences between AF and SR samples. [SupplementalTable_S9.xlsx]

**Table S10**. Subset of candidate loci with proximal DMRs. [SupplementalTable_S10.xlsx]

**Table S11**. RT-QPCR and ChIP-QPCR primers used for targeted validation of candidate regions. [SupplementalTable_S11.xlsx]

**Table S12**. Overlap of chromatin accessibility regions across cardiac cell types with enriched H3K27ac regions and DMRs. [SupplementalTable_S12.xls]

**SUPPLEMENTAL FIGURES**

**Figure S1. Experimental design summary and extended details on H3K27ac profiling** (related to Figure 1)

**a.** Summary of experimental design in the study. Atrial fibrillation (AF) or sinus rhythm (SR) atrial samples from the right (RA) and left atrium (LA) were primarily used for H3K27ac ChIP-sequencing to profile active promoters and enhancers across individuals and sample groups. Most of these samples had been previously used for transcriptome profiling, and we additionally obtained DNA methylation EPIC array data for a subset of samples. See Table S1 for full details.

**b.** Numbers of H3K27ac peaks called in each sample (Methods). Samples are coloured by sample group, with a similar average number of peaks obtained for each (AF-LA: 49,768; AF-RA: 46,421; SR-LA: 53,511; SR-RA: 47,288).

**c.** Peak lengths for each individual sample are represented as box plots, and coloured according to sample group. Average lengths per sample group: AF-LA, 1,189 bp; AF-RA, 1,111; SR-LA, 1,024; SR-RA, 740.

**d.** Barplot with number of peaks consistently called in samples from each group, as obtained with Genrich (“reference peak set”, Methods). Peaks called in one or more input samples from each group were excluded from this set (“greylisted”, grey bars).

**e**. Lengths for reference peaks in each sample group. Average lengths: AF-LA, 991 bp; AF-RA, 905; SR-LA, 763; SR-RA, 669.

**f**. Density plots showing distance to annotated transcriptional start sites (TSSs) for reference peaks in each sample group. As expected, most H3K27ac reference peaks are distal to annotated TSSs.

**Figure S2. Methodological details and extended properties for H3K27ac epigenomic regions enriched across sample groups** (related to Figure 2)

**a.** Summary diagram of the approach used to identify enriched H3K27ac genomic regions in each sample group. Peak calling: H3K27ac ChIP-sequencing data was processed to call regions of enrichment (peaks) in each individual sample. Differential pairwise comparisons: quantitative H3K27ac levels were then compared across each pair of samples to identify sets of regions enriched in either of the two samples for each pair. Consensus sets of enriched regions: a set of enriched H3K27ac regions was then defined in each sample group by merging enriched pairwise sets, requiring each consensus region to be present in at least 15-50% of comparisons (Methods). These sets of enriched regions were then compared, with those overlapping more than one sample group excluded from analysis.

**b**. Boxplot representation for lengths of enriched H3K27ac regions in each sample group. Average lengths: AF-LA, 3,972; AF-RA, 3,488; SR-LA, 2,517; SR-RA, 1,103.

**c**. Distributions of distance to the nearest annotated transcriptional start site (TSS) for enriched H3K27ac regions in each sample group

**d**. Heatmap of gene ontology terms (Biological process) associated with enriched H3K27ac regions in each sample group. P-adjusted values indicate significance of GO category enrichment calculated using a hypergeometric test after FDR correction (enrichGO function from clusterProfiler).

See also Tables S2 and S3.

**Figure S3**. **Additional analyses on H3K27ac-enriched regions across sample groups (overlap with transcription factor motifs, binding events and cardiovascular GWAS variants)** (related to Figure 2)

**a**. Heatmap of transcription factor binding motifs (TFBSs) overrepresented in the DNA sequences of enriched H3K27ac regions for each sample group. Significance levels are illustrated in -log10(FDR) scale. These were estimated based on resemblance with known motif databases using the HOMER enrichment test assuming binomial distribution of motif occurrences.

**b.** Selected transcription factor binding events overrepresented in enriched H3K27ac regions for each sample group are represented as a circle plot. Binding data corresponds to ChIP-sequencing sets reported in cardiac tissue or stem-cell derived cardiomyocytes (iPSC-CM or hESC-CM), as catalogued in Remap 2022. Circle sizes denote the number of overlaps, and colour scale the Q-value of significance (FDR-corrected p-value from Fisher’s Exact Test).

**c.** Cardiovascular GWAS variants over-represented in H3K27ac-enriched regions. Circle plot represents fold enrichment (circle size, log10 fold) and statistical significance (circle shade, -log10 of adjusted p-values, hypergeometric test with BH correction for multiple comparisons) for the enrichment of cardiovascular GWAS variants in H3K27ac-enriched regions across sample groups (AF-LA, AF-RA, SR-LA and SR-RA, x-axis). GWAS variants correspond to all lead variants in the NIHR-EBI GWAS catalog plus proxy variants in strong LD for atrial fibrillation (AF), coronary artery disease (CAD), cardiomyopathies (CM) and QT interval duration (QT).

**Figure S4. Gene expression re-analysis and extended details on candidate loci identified from integration of epigenomic and transcriptomic signals** (related to Figure 3)

**a.** Principal component analysis of gene expression levels across individual samples in AF-LA, AF-RA, SR-LA and SR-RA groups, showing the first two principal components as a scatter plot. Variance across PC1 and PC2 provides a degree of separation across anatomical side (LA samples on the left and RA samples on the right).

**b.** Volcano plots of differentially expressed genes (DEGs) in each anatomical side (LA, 570 upregulated genes; RA, 517 upregulated genes) and disease status (AF, 501 upregulated genes; SR, 744 upregulated genes).

**c.** Volcano plots of differentially expressed genes across disease status, either in left atrium (left plot: AF-LA upregulated, 89 genes; AF-RA upregulated, 118 genes) or right atrium samples (right plot: AF-RA upregulated, 61 genes; SR-RA upregulated, 164 genes). The central Venn diagram corresponds to overlap of genes found to be differentially expressed in each anatomical side.

**d.** Association of H3K27ac enriched regions in each sample group with differentially expressed genes, either for AF vs SR samples (left plot) or for enriched regions / DEGs across all four sample groups (right plot). Numbers in each cell represent overlapping gene loci, and colour shades statistical significance (-log_10_(p-value), Fisher’s exact test).

**e.** Definition of candidate loci with altered gene regulation in AF or SR samples. Venn diagrams represent the overlap between upregulated genes (cyan) and enriched H3K27ac regions (violet), either in SR (left) or AF (right). Grey insets under each Venn diagram detail the candidate loci derived from each comparison.

**f.**  and **g.** Examples of candidate loci from e. **f.** (Left) H3K27ac average fold enrichment over input for each sample group across the *COL23A1* locus, including an SR-enriched H3K27ac region (left, SR-enriched). Promoter-capture HiC data in iPSC-cardiomyocytes supports 3D interactions between this region and a distal region downstream. (Right) *COL23A1* expression levels are upregulated in SR samples, in concordance with the H3K27ac enriched region (***, p < 0.001; DESeq2 adjusted p-values) **g**. (Left) Representation as in f. across the *NPPB* locus, including an AF-enriched H3K27ac region with supporting evidence of chromatin contacts with promoters in this locus. (Right) *NPPB* expression levels are significantly higher in AF samples (*, p < 0.05; DESeq2 adjusted p-values).

See also Tables S4, S5 and S6.

**Figure S5. Extended details and analyses of candidate loci in an independent replication cohort** (related to Figures 4 and 5)

**a.** Summary diagram of the experimental approach used for independent validation of candidate loci. A set of 3 AF and 2 SR samples comprising both left and right atrium (R1 to R5) were used for locus-specific quantitative PCR validation of gene expression levels (RT-QPCR) or H3K27ac enrichment (ChIP-QPCR) (Methods).

**b.** Quality control of independent validation samples by RT-QPCR. Expression levels were assessed for cardiac markers (left: *MYH6*, *NPPA* and *MYH7*, using liver-specific *HNF4A* as a control), a left atrium marker (*PITX2*) and a right atrium marker (*BMP10*). P-values: ***, p < 0.001; ** p < 0.01 (Student’s t-test)

**c.** ChIP-QPCR validation of H3K27ac enrichment across selected AF-enriched candidate regions. For each region, top genome tracks show average H3K27ac fold enrichment over input in each sample group in the discovery cohort, enriched candidate regions (coloured bars, bottom), and the location of QPCR primer pairs for each of the two amplicons assessed per region (e.g. P1a and P1b). Bottom barplots show normalised H3K27ac ChIP enrichment (Methods) for the best performing amplicon and each assessed region across validation samples. P-values correspond to one-sided t-test.

**d.** Representation and details as in c. for ChIP-QPCR validation of H3K27ac enrichment across selected SR-enriched candidate regions. P-values correspond to one-sided t-test.

See also Tables S7 and S8.

**Figure S6. EWAS results and additional properties of DMRs across SR and AF samples** (related to Figure 6)

**a.** Manhattan plot for EWAS analysis of EPIC v2 array data across SR and AF samples. Dashed red lines indicate the statistical threshold for genome-wide significance (p < 10^-8^), and labelled points above and below this line correspond to CpGs with methylation levels significantly higher in AF (above) or SR samples (below). Boxplots on the right provide two examples of genome-wide differentially methylated CpGs (DMCs) for the *WWOX* and *LRRC32* loci (respectively).

**b.** Association of differentially-methylated regions (DMRs) with H3K27ac enriched regions and differentially expressed genes in AF and SR samples. (Left) Heatmaps show the number of gene loci associated to both DMRs and H3K27ac enriched regions (top), or DMRs and differentially expressed genes (bottom). Colour scales correspond to statistical significance (-log_10_(p-value), Fisher’s exact test). (Right) Venn diagram showing the overlap of gene loci associated to enriched H3K27ac regions, differentially-expressed genes (DEGs) and DMRs (p=0; Chi-square test of independence). In all cases, gene loci correspond to genes associated to differential regions between AF and SR samples (e.g. both AF-upregulated and SR-upregulated genes).

**c.** Distribution of distances to the nearest annotated TSS for DMRs hypermethylated (red) or hypomethylated (blue) in AF samples.

**d.** Annotation of DMRs according to CpG island annotations (CGIs; Methods), as either within (brown) or outside (orange) CGIs.

See also Tables S9 and S10.

**Figure S7. Additional details and analyses for DMRs and their overlap with transcription factor motifs, binding events and GWAS variants** (related to Figure 7)

**a.** Example of a hypermethylated DMR in the SETMAR locus (DMR49), for differentially-methylated CpGs overlap a number of transcription factor motifs.

*(Top tracks)* Gene and DMR annotation in this genomic locus.

*(Bottom right inset)* DNA methylation differences across the DMR, shown as beta-values for AF (sienna) and SR (cyan) samples. **: p-value < 0.01; ***: p-value < 0.001. Annotations below the plot correspond to individual CpGs and the sequence motifs for MNT, ARNT, SOX8, IRF6 and ZBED2 (green boxes). Sequence logos are shown for each of these transcription factors, along with expression levels of *MNT* in AF and SR samples (bottom boxplot)

*(Bottom left boxplots)* Expression levels of *SETMAR* are significantly higher in SR patients (*: p < 0.05), suggesting hypermethylation of the DMR in AF patients may reduce *SETMAR* expression. Expression levels of *SUMF1* are similar in SR and AF samples.

**b.** Gene expression levels in AF (sienna) and SR samples (cyan) for genes in the *LRRC4B* / *STY3* / *SHANK1* locus. Only *LRRC4B* expression is significantly different between control and AF patients (****: p < 0.0001; n.s.: non-significant differences). Details of annotation tracks as in Figure 7.

**c.** Cardiovascular GWAS variants over-represented in DMRs. Circle plot represents fold enrichment (circle size, log10 fold) and statistical significance (circle shade, -log10 of adjusted p-values, hypergeometric test with BH correction for multiple comparisons) for the enrichment of cardiovascular GWAS variants in all, hypermethylated or hypomethylated DMRs. GWAS variants correspond to all lead variants in the NIHR-EBI GWAS catalog plus proxy variants in strong LD for atrial fibrillation (AF), coronary artery disease (CAD), cardiomyopathies (CM) and QT interval duration (QT).

**d.** Selected transcription factor binding events overrepresented in DMRs (hyper- or hypomethylated) are represented as a circle plot. Binding data corresponds to ChIP-sequencing sets reported in cardiac tissue or stem cell-derived cardiomyocytes (iPSC-CM and ESC-CM), as catalogued in Remap 2022. Circle sizes denote the number of overlaps, and colour scale the Q-value of significance (FDR-corrected p-value from Fisher’s Exact Test).

See also Table S9.

**Table S7.** Candidate loci selected for validation in an independent replication cohort with locus-specific quantitative PCR.


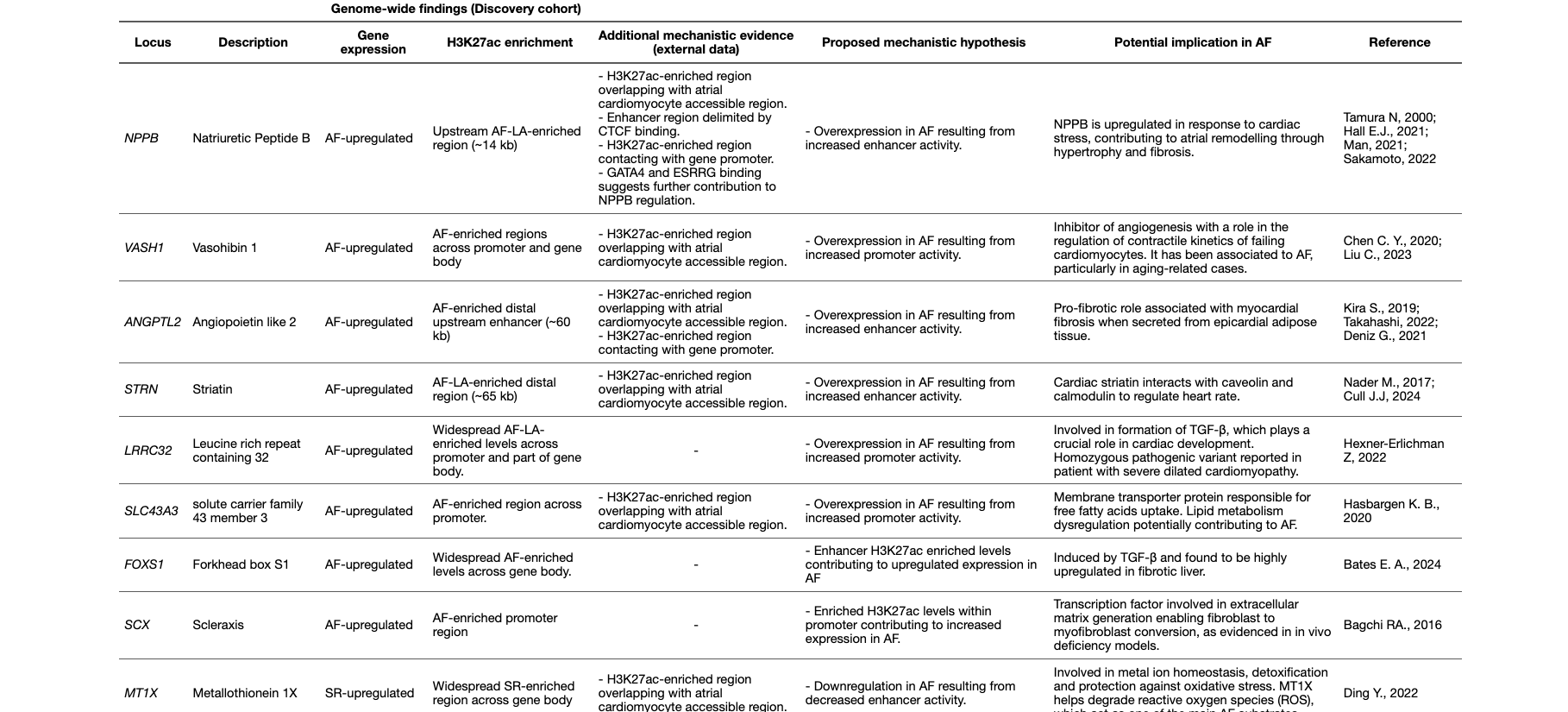

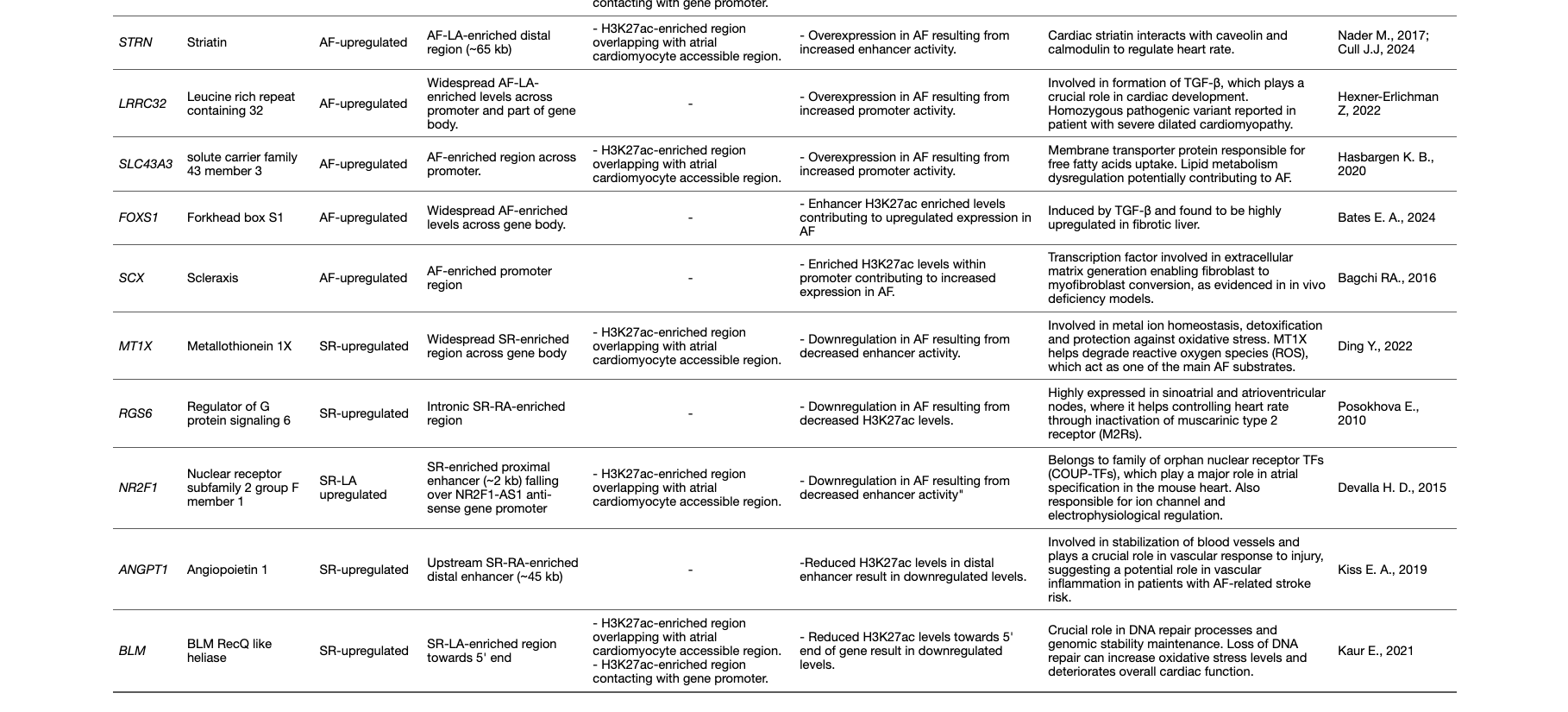

Supplement: Supplementary file 1 — Supplementary Material 1 [file 13148_2026_2076_MOESM1_ESM.docx]
